# E2ENNet: An end-to-end neural network for emotional brain-computer interface

**DOI:** 10.3389/fncom.2022.942979

**Published:** 2022-08-12

**Authors:** Zhichao Han, Hongli Chang, Xiaoyan Zhou, Jihao Wang, Lili Wang, Yongbin Shao

**Affiliations:** ^1^School of Electronic and Information Engineering, Nanjing University of Information Science and Technology, Nanjing, China; ^2^The Key Laboratory of Child Development and Learning Science of Ministry of Education, Southeast University, Southeast University, Nanjing, China

**Keywords:** electroencephalogram (EEG), neurocognitive, emotional brain-computer interface, depthwise separable convolution, long short-term memory

## Abstract

**Objectve:**

Emotional brain-computer interface can recognize or regulate human emotions for workload detection and auxiliary diagnosis of mental illness. However, the existing EEG emotion recognition is carried out step by step in feature engineering and classification, resulting in high engineering complexity and limiting practical applications in traditional EEG emotion recognition tasks. We propose an end-to-end neural network, i.e., E2ENNet.

**Methods:**

Baseline removal and sliding window slice used for preprocessing of the raw EEG signal, convolution blocks extracted features, LSTM network obtained the correlations of features, and the softmax function classified emotions.

**Results:**

Extensive experiments in subject-dependent experimental protocol are conducted to evaluate the performance of the proposed E2ENNet, achieves state-of-the-art accuracy on three public datasets, i.e., 96.28% of 2-category experiment on DEAP dataset, 98.1% of 2-category experiment on DREAMER dataset, and 41.73% of 7-category experiment on MPED dataset.

**Conclusion:**

Experimental results show that E2ENNet can directly extract more discriminative features from raw EEG signals.

**Significance:**

This study provides a methodology for implementing a plug-and-play emotional brain-computer interface system.

## 1. Introduction

Emotion is the basis of daily human life and plays an essential role in human cognitive functions, rational decisions, and interpersonal communications (Waldron, 1994; Picard et al., 2001; Martinovski and Mao, 2009). It is extremely important to identify emotions accurately especially in the field of brain-computer interaction (Cowie et al., 2002; Jin et al., 2020, 2021). Automatic emotion recognition technology is introduced to human-computer interaction, which can remarkably improve the quality of user experience and enhance the interactions between computer and humanity (Stamos and Naeem, 2017).

There are two reflections of emotion including external and internal reactions: external reactions include human facial expressions, gestures, or speeches; internal reactions include skin electrical responses, heart rate, blood pressure, respiratory rate, electroencephalogram (EEG), electroencephalography (EOG) (Yu et al., 2019), magnetoencephalogram (MEG) (Christian et al., 2014). From the perspective of neuroscience (Lotfi and Akbarzadeh-T., 2014), the main areas of the cerebral cortex are closely related to human emotions (Britton et al., 2006; Etkin et al., 2011; Lindquist and Barrett, 2012), which inspires us to record the neural activities of the brains by putting EEG electrodes on the scalp to collect EEG signals to recognize human emotions.

**Figure 1:**
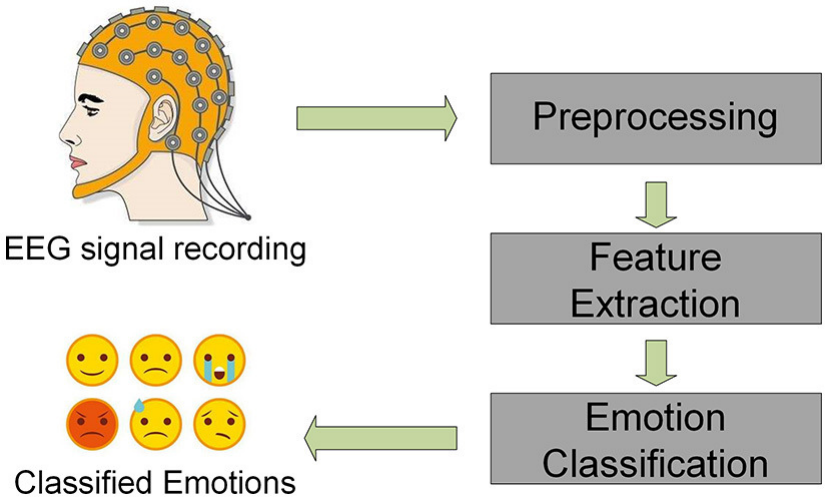
Traditional framework of EEG emotion recognition.

EEG signal contains emotional information, which has been widely used in the field of emotion recognition in recent years (Soroush et al., 2017; Sulthan et al., 2018; Alarcao and Fonseca, 2019). In traditional EEG emotion recogniton process, feature extraction is a vital procedure. As shown in Figure 1, after preprocessing the EEG signals, usually it is necessary to extract features from raw EEG signals, then input them into the network for classification and recognition (Duan et al., 2013; Chen et al., 2021; Ma et al., 2021). Duan et al. (2013) proposed the differential entropy (DE) feature of five frequency bands and obtained satisfactory classification results using DE features. Li et al. (2019) used short-time Fourier transform to extract time-frequency features, calculated the power spectral density (PSD) features in theta, alpha, beta, and gamma bands, and used LSTM to discriminate emotions, which achieved significant classification results. Ma et al. (2021) proposed a Beetle Antenna Search (BAS) algorithm that extracted three different features in three different bands and six channels and an SVM classifier was applied for classification. Compared with traditional SVM methods, the classification accuracy of the BAS-SVM method has gained a 12.89% enhancement. In recent years, deep learning methods are widely used in emotion recogniton (Jia et al., 2020a; Li et al., 2020; Zhou et al., 2021). Song et al. (2018) designed DE features based on electrode positions and used graph convolutional neural network (GCNN) as a classifier. Zhang et al. (2019) innovatively combined DE features extracted from the EEG dataset with the features extracted from the facial expression dataset and constructed a spatial-temporal recurrent neural network (STRNN) for emotion recognition. Li et al. (2018) proposed a bi-hemisphere domain adversarial neural network (BiDANN), which used DE as the input feature and conducted both subject-dependent and subject-independent experiments on the SEED dataset, achieving relatively state-of-the-art performance. Hao et al. (2021) proposed a lightweight convolutional neural network that extracts PSD features as input and conducted experiments on the DEAP dataset, which attained 82.33 and 75.46% for Valance and Arousal, respectively. Chen et al. (2021) proposed an integrated capsule convolution neural network (CapsNet), which used Wavelet packet transform (WPT) for feature extraction. The average accuracy of the two-category and four-category experiments on DEAP has reached 95.11 and 92.43%, respectively.

On the other hand, many deep learning methods need not to extract features manually while run end-to-end. Alhagry et al. (2017) proposed an end-to-end deep learning neural network to identify emotions from original EEG signals. This network used LSTM-RNN to learn features from EEG signals and a softmax classifier used for emotion recognition. However, they ignored the vital factor of the spatial relationship between electrodes. Yang et al. (2018) proposed a parallel convolution recurrent neural network to extract spatial features of EEG signals, which achieved acceptable results in emotion recognition tasks but ignored the point of temporal correlations. During the procedure, it may also lose some features that contain fruitful emotional information. It is still a worthy topic that how to design a practical deep learning framework to recognize and classify emotions from the original EEG signals directly. EEGNet (Lawhern et al., 2016) is a compact convolutional neural network suitable for EEG signals. Our study introduced extracting EEG features and classifying emotions by using depthwise separable convolution. Due to the solid internal relationship between different channels of EEG signal and the time correlations. Inspired by Lawhern et al. (2016), we proposed an end-to-end neural network (E2ENNet) for EEG emotion, which concatenates EEGNet and LSTM (Long-Short Term Memory). We use depthwise separable convolution to extract features from multi-channel original EEG signals, LSTM for searching the correlations between those features. Finally, a softmax classifier is applied to output the classification results.

We evaluated the proposed model on three public datasets, i.e., DEAP (Koelstra, 2012), DREAMER (Stamos and Naeem, 2017), and MPED (Song et al., 2019), achieveing state-of-the-art accuracy among existing methods. The main contributions of this paper are as follows:

- We proposed E2ENNet for EEG emotion recognition. This network combined EEGNet and LSTM, which simultaneously considerd the spatial information and the time correlations in EEG signals. At the same time, it avoided the complicated manual feature extraction and made full use of all information in raw EEG signals, which realized end-to-end EEG emotion recognition.
- We conducted extensive subject-dependent experiments on three public datasets: DEAP, DREAMER, MPED. The average accuracy of two-category classification is 96.25% (Valance) and 96.16% (Arousal) on the DEAP dataset; the average accuracy of two-category classification is 97.84% (Valance), 98.31% (Arousal), and 98.64% (Dominance) on the DREAMER dataset; it also achieved an average accuracy of 41.73% for the seven-category on the MPED dataset. Experimental results demonstrate that the proposed method has achived state-of-the-art performance on emotion recognition among other deep learning methods.

The remainder of this paper is organized as follows. Section 2 presents the proposed method, E2ENNet. Section 3 discusses extensive experiments on three different public datasets. Finally, a conclusion is given in Section 4.

## 2. Proposed method

**Figure 2:**
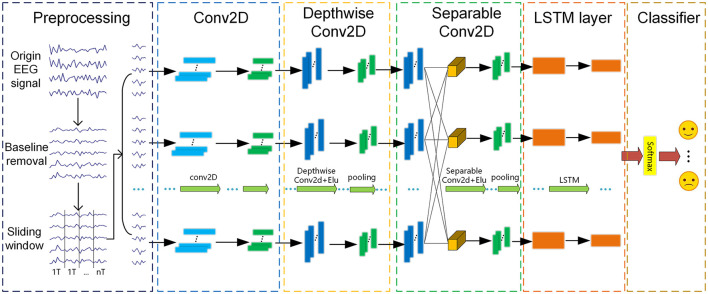
The structure diagram of end-to-end neural network on EEG-based emotion recognition.

This section mainly introduces our proposed end-to-end method, i.e., E2ENNet, including preprocessing, Conv2D, DepthwiseConv2D, Separable Conv2D (Howard et al., 2017), LSTM layer and classifier as shown in Figure 2. Spatial and temporal pieces of information are extracted as emotion features for EEG emotion recognition. Firstly, we removed the baseline and used a sliding window to divide the signal into segments with a duration of 1s. Then, these segments are sequentially fed to Conv2D, DepthwiseConv2D, SeparableConv2D, LSTM layer in order to extract spatial and temporal features. Finally, a softmax function is used to classify the extracted features. The ultimate result of the experiment has a remarkable increase due to the end-to-end neural network.

### 2.1. Preprocessing

There are two parts of preprocessing, i.e., baseline removal and sliding window slicing. Generally, EEG signals obtained by video evoked material stimulation include baseline signals and test signals (Koelstra, 2012; Stamos and Naeem, 2017). Yang et al. (2018) mentioned that baseline removal can improve the recognition accuracy of EEG signals on the DEAP dataset.

For every single trial, let ${\text{C}}_{R}=\left[{\text{C}}_{B},{\text{C}}_{T}\right]\in {\mathbb{R}}^{M\times N}$ represents the collected EEG signal with sampling frequency of *H* Hz and duration of *T*_1_ s. *M* is the number of EEG electrodes, *N* is the collected sample points. Let ${\text{C}}_{B}\in {\mathbb{R}}^{M\times L}$ represents the baseline signals with duration of *T*_2_ s and *L* sample points. **c**_*i*_(*i* = 1, 2, …, *T*_2_) represents the baseline signal in the *i*-th second. Therefore, the average value of baseline signal per second can be expressed as:

(1) $$\begin{array}{r} \bar{{\text{C}}_{B}}=\frac{\sum_{i=1}^{T_{2}}{\text{c}}_{i}}{T_{2}} \end{array}$$

where $\bar{{\text{C}}_{B}}\in {\mathbb{R}}^{M\times H}$ represents the average value of the baseline signal per second.

Let ${\text{C}}_{T}\in {\mathbb{R}}^{M\times J}$ represents the test signal, duration is *T*_3_ s, *J* represents the number of sample points. For removing the baseline in the test signal, divide it into several non overlapping slices **c**_*j*_(*j* = 1, 2, …, *T*_3_) with a 1-s time window. Therefore, the baseline removed signal per second can be expressed as:

(2) $$\begin{array}{r} {{\text{c}}_{j}}^{{}^{\prime }}={\text{c}}_{j}-\bar{{\text{C}}_{B}} \end{array}$$

Finally, put these baseline removed slice signals ${{\text{c}}_{j}}^{{}^{\prime }}$ into a new matrix ${\text{C}}_{T}\in {\mathbb{R}}^{M\times J}$.

Furthermore, in order to increase the number of samples of EEG experiment data. An EEG test signal ${\text{C}}_{T}\in {\mathbb{R}}^{M\times J}$ is usually transmitted by sliding window technology into several non overlapping samples **s** = **s**_1_, **s**_2_, …, **s**_*n*_. Where **s**_*i*_(*i* = 1, 2, …, *n*) represents the *i*-th sample, *T* represents the sample point of each sliding window. In this paper we use 1-s time window to silice the EEG signals, i.e., *T* = *H*.

### 2.2. The framework of E2ENNet

In this paper, the preprocessed EEG signals do not need to extract features manually and can directly become the input of the E2ENNet model for emotion recognition. E2ENNet is formated by four blocks, i.e., a 2D convolution block, a depthwise convolution block, a depthwise separable convolution block and a LSTM block. EEG features can be extracted effectively from the original EEG signals through the convolution blocks. The E2ENNet we proposed adds an LSTM module behind the convolution blocks, composed of two Reshape layers and two LSTM layers. It can make up for the lack of exploring the channel-wise correlations of EEG signals. Finally, a fully connected layer is applied to combine those features and input them into a softmax classifier for emotion classification. Table 1 shows the detailed parameters of the E2ENNet module, where *C* represents the channels of EEG, *T* is the number of the sample point, *F*_1_ is the number of depthwise convolution kernels, *F*_2_ is the number of pointwise convolution kernels, *N* is the number of categories of classification.

**Table 1:** Detailed parameters of E2ENNet model.

**Block**	**Layer**	**Size**	**Output**	**Activation function**
1^*^	Input		(C,T)	
	Reshape		(C,T,1)	
	Conv2D	(1,64)	(C,T,*F*_1_)	Linear
	BatchNorm		(C,T,*F*_1_)	
2^*^	DepthwiseConv2D	(C,1)	(1,T,2 × *F*_1_)	Linear
	Batchnorm		(1,T,2 × *F*_1_)	
	Activation		(1,T,2 × *F*_1_)	Elu
	AveragePool2D	(1,4)	(1,T/4,2 × *F*_1_)	
	Dropout		(1,T/4,2 × *F*_1_)	
3^*^	SeparableConv2D	(1,16)	(1,T/4,*F*_2_)	Linear
	Batchnorm		(1,T/4,*F*_2_)	
	Activation		(1,T/4,*F*_2_)	Elu
	AveragePool2D	(1,8)	(1,T/32,*F*_2_)	
	Dropout		(1,T/32,*F*_2_)	
4^*^	Reshape		(*F*_2_ × (*T*/32),1)	
	LSTM	64	64	
	Reshape		(64,1)	
	LSTM	32	32	
Classifier	Dense		N	Softmax

* Block1-4 represents the 2D convolution block, depthwise Convolution block, depthwise separable convolution block and LSTM block, respectively.

E2ENNet is a lightweight convolution neural network. The core idea is to use depthwise separable convolution to extract EEG features and LSTM to search for the relationship between those features. Depthwise separable convolution divides a standard convolution operation into two steps: depthwise convolution and pointwise convolution. For depthwise convolution, the number of convolution kernels is the same as the number of input feature maps. Each kernel is convoluted separately corresponding to a channel, that is, the same number of feature maps as the input feature maps are generated. However, this operation completes after each channel of the input layer is convolved independently, but the information of different feature maps in the same space can not be made full use of. Therefore, the pointwise convolution is introduced, which combines these different feature maps to generate a new feature map. The pointwise convolution operation is very similar to the conventional convolution. Except that the size of the convolution kernel is 1 × 1 × *M*, *M* is the number of feature maps of the previous layer. It combines the results of depthwise convolution to generate a brand new feature map. The number of convolution kernels is equal to the number of feature maps. Depthwise separable convolution greatly reduces the amount of calculation and model depth of the neural network. However, its classification accuracy is not lower than the traditional CNN model (Tan and Le, 2019).

In the standard convolution layer, it is assumed that for feature map *F*, the format of input EEG signal is *S*_*f*_ × *S*_*f*_ × *M*, the standard convolution of the convolution kernel *K* is *S*_*k*_ × *S*_*k*_ × *M* × *N*. The format of output feature map *G* is *S*_*g*_ × *S*_*g*_ × *N*. The operation of standard convolution is shown as Equation (3):

(3) $$\begin{array}{r} G_{k,l,n}=\underset{i,j,m}{\sum }K_{i,j,m,n}F_{k+i-1,l+j-1,m} \end{array}$$

Assuming that the number of input channels is *M* and the number of output channels is *N*, the calculation amount of standard convolution is: *S*_*k*_ × *S*_*k*_ × *M* × *N* × *S*_*f*_ × *S*_*f*_. A standard covolution *S*_*k*_ × *S*_*k*_ × *M* × *N* can be decomposed into two steps: depthwise convolution and pointwise convolution. These two steps add up to form a full depthwise separable convolution. The function of depthwise convolution is filtering, in which the format is *S*_*k*_ × *S*_*k*_ × 1 × *M*, and the output format is *S*_*g*_ × *S*_*g*_ × *M*. The function of pointwise convolution is channel combination, the format is 1 × 1 × *M* × *N*, and the output format is *S*_*g*_ × *S*_*g*_ × *N*. A complete depthwise separable convolution is expressed as Equation (4):

(4) $$\begin{array}{r} \hat{G_{k,l,n}}=\underset{i,j}{\sum }\hat{K_{i,j,m,n}}F_{k+i-1,l+j-1,m} \end{array}$$

Where $\hat{K}$ is the kernel of depthwise convolution, the size is *S*_*k*_ × *S*_*k*_ × *M*. Apply the *m*-th kernel of $\hat{K}$ to the *m*-th channel of *F*. We can get the *m*-th channel of filtered feature map $\hat{G}$. The number of input channels is *M*, the amount of calculation of depthwise convolution is *S*_*k*_ × *S*_*k*_ × *M* × *S*_*f*_ × *S*_*f*_. The amount of calculation of a complete depthwise separable convolution is *S*_*k*_ × *S*_*k*_ × *M* × *S*_*f*_ × *S*_*f*_ + *M* × *N* × *S*_*f*_ × *S*_*f*_. It is $\frac{1}{N}+\frac{1}{S_{k}^{2}}$ as the calculation of standard convolution.

In E2ENNet, as the network deepens, the amount of parameters also grows exponentially. The sensitivity of divergent information to the non-normalized network decreases. Therefore, we use batch normalization (Liu et al., 2018) to normalize the output. The normalization function is defined as follows:

(5) $$\begin{array}{r} BN\left(X_{i}\right)=\frac{\left(X_{i}-E\left(X_{i}\right)\right)}{\sqrt{Var\left(X_{i}\right)}} \end{array}$$

Where *E*(*X*_*i*_) is the average value of neuron *X*_*i*_ in each batch of training data, and the denominator is the standard deviation of neuron *X*_*i*_' activation in each batch of training data. The features are reconstructed to avoid affecting the feature distribution learned by this layer of the network:

(6) $$\begin{array}{r} E\left(X_{i}\right)=\frac{1}{m}\overset{L}{\underset{i=1}{\sum }}X_{i} \end{array}$$

(7) $$\begin{array}{r} Var\left(X_{i}\right)=\frac{1}{m}\overset{L}{\underset{i=1}{\sum }}{\left[X_{i}-E\left(X_{i}\right)\right]}^{2} \end{array}$$

In E2ENNet, the batch normalization technique is used to normalize the features learned in the convolution blocks to get a (0,1) normal distribution.

To further figure out the relationship between multi-channels of time-series, LSTM network (Hochreiter and Schmidhuber, 1997) is introduced in this paper. LSTM plays a critical role in processing time-series signals to selectively learn information about them. It is also widely used in the field of EEG emotion recognition (Alhagry et al., 2017; Zhang et al., 2019). In the traditional neural network methods, the inputs are independent, so they ignore sequence information. RNN (Jain et al., 2016) is very effective for data with sequence characteristics, it can mine time series information in the data (Wang et al., 2016). Long short-term memory is a special kind of RNN that can solve the problem of vanishing gradients and easily learn long-term dependent information. Therefore, we use the normalized features *f*_*B*_*N* = *BN*(*X*_*i*_) as the input of LSTM. Let *i*_*t*_, *g*_*t*_, *c*_*t*_, *o*_*t*_ be the input gate, forget gate, cell activation, and output gate of LSTM, respectively. The calculation process is expressed as the following equation:

(8) $$\begin{array}{l} \{\begin{array}{l} i_{t}=\sigma \left(W_{x}if_{a}t+W_{h}ih_{t-1}+W_{c}ic_{t-1}+b_{i}\right)\\ g_{t}=\sigma \left(W_{x}gf_{a}t+W_{h}gh_{t-1}+W_{c}gc_{t-1}+b_{g}\right)\\ c_{t}=g_{t}c_{t-1}+i_{t}tanh\left(W_{x}cf_{a}t+W_{h}ch_{t-1}+b_{c}\right)\\ o_{t}=\sigma \left(W_{x}of_{a}t+W_{h}oh_{t-1}+W_{c}oc_{t-1}+b_{o}\right)\\ h_{t}=o_{t}tanh\left(c_{t}\right) \end{array} \end{array}$$

Where σ represents the sigmoid function, *h*_*t*_ represents the hidden vector of LSTM cell unit, *W*_*xi*_, *W*_*hi*_, *W*_*ci*_, *W*_*xg*_, *W*_*hg*_, *W*_*cg*_, *W*_*xc*_, *W*_*hc*_, *W*_*xo*_, *W*_*ho*_ and *W*_*co*_ are parameters of the model. Finally, as the last part of E2ENNet, the softmax layer is used as the classifier. Use the output *H* = [*h*_1_, *h*_2_, …, *h*_*n*_] of LSTM as the input of softmax to recognize emotions, as following equation:

(9) $$\begin{array}{r} P=softmax\left(\omega H+b\right) \end{array}$$

Where *P* = *P*_1_, *P*_2_, …, *P*_*n*_,*P*_*i*_(*i* = 1, 2, …, *n*) represents the prediction probability of the *i*-th EEG sample. ω and *b* are the wight term and offset term. Finally, calculate the cross-entropy error of all data that has already been labeled.

(10) $$\begin{array}{r} \tau =-\overset{n}{\underset{i=1}{\sum }}\hat{S_{i}}log\left(P_{i}\right) \end{array}$$

Where $\hat{S_{i}}$ is the label of the *i*-th EEG sample. When the cross-entropy loss decreases, the accuracy of emotion recognition increases.

## 3. Experiments and results

### 3.1. Introduction of datasets

The E2ENNet model we proposed has been tested on three public datasets: DEAP, DREAMER, and MPED. As shown in Table 2. Here are details of three datasets below:

- **DEAP:** DEAP dataset is a multimodal emotion dataset containing a variety of physiological signals, which was proposed by the research team of Queen Mary University in London. The dataset contains 40 music videos watched by 32 subjects. EEG and other physiological signals were recorded. In this experiment, the sampling frequency of EEG signals is reduced to 128 Hz, and the EOG artifact is removed by blind source separation technology. After pretreatment of each experiment, the EEG data includes 60 s of test data and 3 s of baseline data. Subjects were asked to record and evaluate each video with a value of 1–9 in Valance, Arousal, Dominance, and liking. We selected Valance and Arousal in the two-category experiment as the evaluation criteria. The threshold was set to 5, which was divided into High/Low Valance and High/Low Arousal.
- **DREAMER:** DREAMER dataset is a multimodal dataset collected by the research team of the University of Western Scotland, including EEG and ECG signals. Twenty-three subjects watched 18 videos and were asked to record the Valance, Arousal, and Dominance after each stimulus. EEG signals were recorded using Emotiv EPOC equipment with a sampling frequency of 128 Hz. The length of the video is 65–393 s. All EEG data were edited to 61 s in this experiment, including 60 s of test data and 1 s baseline data. Besides, most artifacts (eye electricity, eye movement, heartbeat interference, etc.) have been removed by the FIRS filter. We selected Valance, Arousal, and Dominance as the evaluation criteria. The label range is 1–5, and 3 was chosen as the threshold, which is divided into High/Low Valance, Arousal, and Dominance.
- **MPED:** MPED dataset is a large open-source emotional dataset collected by the Wenming Zheng team of Southeast University, China, which contains four physical signals: EEG, skin electricity, respiratory, and ECG data. The dataset contains 28 Chinese videos watched by 23 subjects. The video includes joy, fun, neutrality, sadness, fear, disgust, and anger. There are seven types of emotions, each type of emotion has four video clips. The acquisition equipment is an ESI Neuroscan with 62 electrodes and a sampling frequency of 1,000 Hz. The data we use in this experiment has already removed noise interference, downsampled to 128 Hz, and only contains the data of EEG signal. The EEG data is clipped to 120 s and does not contain a baseline signal.

**Table 2:** Details of three different datasets.

**Dataset**	**Electrodes**	**Evaluation criterion**
DEAP	32	Two-category: High/Low Valance,High/Low Arousals
DREAMER	14	Two-category: High/Low Valance,Arousal,Dominance
MPED	62	Seven-category: joy,fun,neutrality,sadness, fear,disgust,anger

### 3.2. Experiment environment and settings

E2ENNet model and preprocessing are implemented based on Python3.8 under the Keras framework. The experimental environment is Inter(R)Core(TM)i5-10400CPU@2.90Hz, 16GB memory, NVIDIA Geforce GTX1060 6G graphics, 64 bit Windows 10 system. All experiments on the database are subject-dependent experiments, i.e., the train set an test set come from one subject. The number of EEG channels *C* is set to 32, 14, and 62 for DEAP, DREAMER, and MPED datasets, respectively. For the number of samples, points *T* are all set to 128 according to the sampling frequency. *F*_1_ and *F*_2_ are set to 8 and 16, respectively. For the number of categories *N*, DEAP and DREAMER two-category experiments are set to 2, and MPED seven-category experiments are set to 7. Adam optimizer is used to optimize the training process. The learning rate is 0.005, the batch size is 16, and the number of iterations is 200.

### 3.3. Experiments on three public datasets

#### 3.3.1. Experiments on DEAP dataset

The format of original data is 40 × 32 × 8064, 40 represents 40 trials, 32 represents 32 electrodes used in EEG, duration of each video *T*_1_ = 63*s*, sampling frequency is 128Hz, baseline signal *T*_2_ = 3*s*. Set the average value of baseline signal for the first 3 s as $\bar{{\text{C}}_{B}}$. Then subtract the average value of baseline signal per second $\bar{{\text{C}}_{B}}$ from the test signal for the last 60 s *T*_3_. The baseline removed slice signal is obtained as experimental data. Moreover, use 1 s non overlapping window to slice the experimental data. Sixty segments were obtained in each trail. We do all experiments under the subject-dependent experimental protocol. Each subject gets 60 × 40 = 2,400 samples, the format of each sample is 32 × 128. We divide the data of 40 trials into the training set and testing set according to the ratio of 4:1, i.e., 32 trials are used as the training set, and 8 trials are used as the testing set. 8 trials of the training set are randomly selected as the validation set. The segmentation is performed five times until every single trial has been trained and tested.A 5-fold cross-validation dataset is constructed, take the average accuracy of five experiments as the final experimental result.

We construct two traditional classification algorithms, SVM and DBN, based on differential entropy (DE) features to verify the model's effectiveness. Both methods go through the same preprocessing steps as the E2ENNet model, namely baseline removal and sliding window slice. According to the method of Duan et al. (2013). DE features on five frequency bands: Delta (1–3 Hz), Theta (4–7 Hz), Alpha (8–13 Hz), Beta (13–30 Hz), and Gamma (30–45 Hz) are extracted as the input of SVM and DBN algorithms. And three other state-of-the-art classification methods are compared:

- ECLGCNN (Yin et al., 2021): extract DE features from preprocessed EEG signals to build cubes, and classify them by fusion model of graph convolution neural network and LSTM.
- ACRNN (Tao et al., 2020): a convolution recurrent neural network based on an attention mechanism is proposed, which fully considers the weights of different EEG channels and the spatial information in EEG signals.
- CapsNet (Chen et al., 2021): wavelet packet transform (WPT) is used to extract features, and an integrated capsule network is used as the classifier for EEG emotion classification.

**Figure 3:**
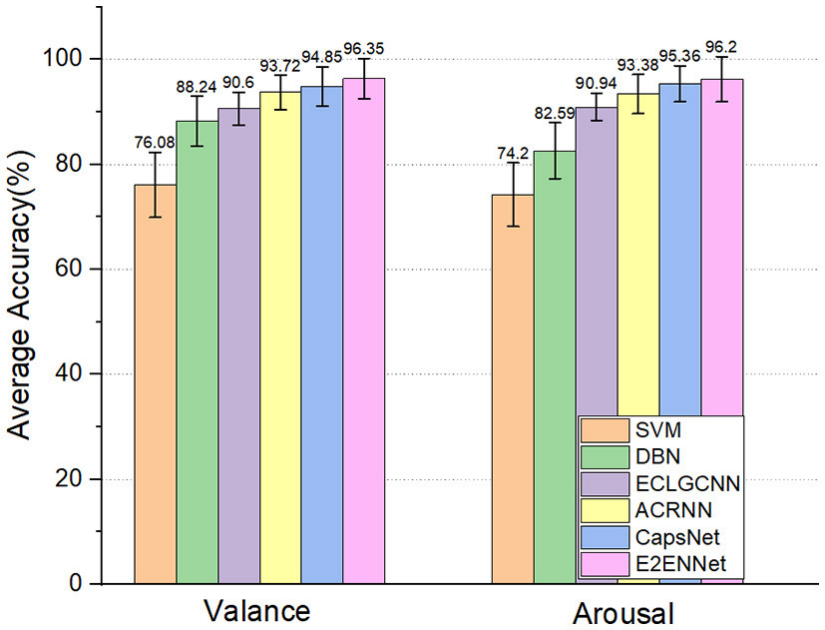
Two-category classification comparison experiment on DEAP dataset.

As shown in Figure 3, we can see that the deep learning method performs better than the two machine learning methods. This shows that deep learning methods can better capture the features in EEG signals for emotion recognition. Compared with other deep learning methods, the E2ENNet model has achieved an average classification accuracy of 96.35% and 96.2% in the dimension of Valance and Arousal, respectively, in the emotion classification experiment on the DEAP dataset, which is higher than the traditional machine learning methods and the above classification methods.

#### 3.3.2. Experiments on DREAMER dataset

The format of original data is 18 × 14 × 7,808, 18 represents 18 trials, 14 represents 14 electrodes used in EEG, duration of each video *T*_1_ = 61*s*, sampling frequency is 128 Hz, baseline signal *T*_2_ = 1*s*. The baseline signal of the first second is $\bar{{\text{C}}_{B}}$, then subtract the value of baseline signal $\bar{{\text{C}}_{B}}$ from the test signal for the last 60 s *T*_3_. The baseline removed slice signal is obtained as experimental data. Furthermore, use 1 s non overlapping window to slice the experimental data. Sixty segments were obtained in each trial. We do all experiments under the subject-dependent experimental protocol. Each subject gets 60 × 18 = 1,080 samples, the format of each sample is 14 × 128. We divide the data of 18 trials into the training set and testing set according to the ratio of 5:1, i.e., 15 trials are used as the training set, and 3 trials are used as the testing set. 3 trials of the training set are randomly selected as the validation set. The segmentation is performed six times until every single trial has been trained and tested. A 6-fold cross-validation dataset is constructed, take the average accuracy of six experiments as the final experimental result.

To verify the effectiveness of the E2ENNet model, consistent with Section 3.3.1. We compare the experimental results with SVM and other deep learning methods:

- DGCNN (Song et al., 2018): A graph representation method for multi-channel EEG data. Constructs the connection relationship between each vertex node of the graph by learning the adjacency matrix, use DE and other features to classify emotions.

**Figure 4:**
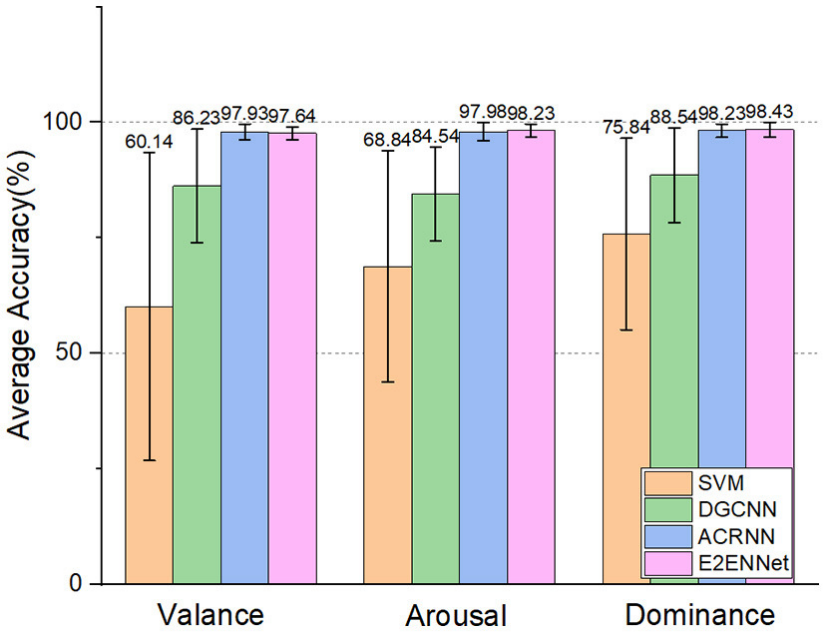
Two-category classification comparison experiment on DREAMER dataset.

And ACRNN (Tao et al., 2020) on three emotional dimensions (Valance, Arousal, and Dominance). As shown in Figure 4, we can see:

1. E2ENNet model achieved 97.64, 98.23, and 98.42% accuracy in Valance, Arousal, and Dominance dimensions, respectively. Among them, the accuracy of Arousal and Dominance is the highest among the four methods.
2. The accuracy of the Valance dimension is a little lower than that of the ACRNN model, probably because its classification accuracy has reached the bottleneck.

#### 3.3.3. Experiments on MPED dataset

The format of original data is 28 × 62 × 1,5360, 28 represents 28 trials, 62 represents 62 electrodes used in EEG, duration of each video *T*_1_ = 120*s*, sampling frequency is downsampled to 128Hz. Thus the EEG data used in this article has already been removed baseline. There is no need to remove the baseline again, and the data can be directly obtained as experimental data. Furthermore, use 1 s no overlapping window to slice the experimental data. One hundred and twenty segments were obtained in each trial. Then each subject gets 120 × 28 = 3,360 samples, the format of each sample is 62 × 128. According to the experimental protocol three of Song et al. (2019)'s. The data of 21 trails are selected as the training set, and 7 trials are selected as the testing set to ensure the samples of 7 emotions in the training set and testing set are balanced. i.e., the sample ratio of the training set and testing set is 3:1, and 7 trials of the training set are randomly selected as the validation set. The segmentation is performed four times until every single trial has been trained and tested. Take the average accuracy of four experiments as the final experimental result.

In order to verify the performance of the model under multi-classes classification tasks. We compare the experimental results with SVM (Song et al., 2019), and other state-of-the-art deep learning methods in subject-dependent experimental protocol:

- A-LSTM (Song et al., 2019): adding attention mechanism to the LSTM network, extracting discriminative features by focusing on the temporal information of time series to classify emotions.
- DANN (Ganin et al., 2016): drawing on the idea of adversarial learning, classify target domain data with source domain data.
- BiDANN (Li et al., 2021): this method takes into account the distribution difference between training and testing data and the asymmetry of the left and right hemispheres of the brain, using DE features to classify emotions.
- SGA-LSTM (Liu et al., 2019): using attention mechanism and combining GCNN with LSTM to focus on specific EEG channels for emotion recognition.

**Figure 5:**
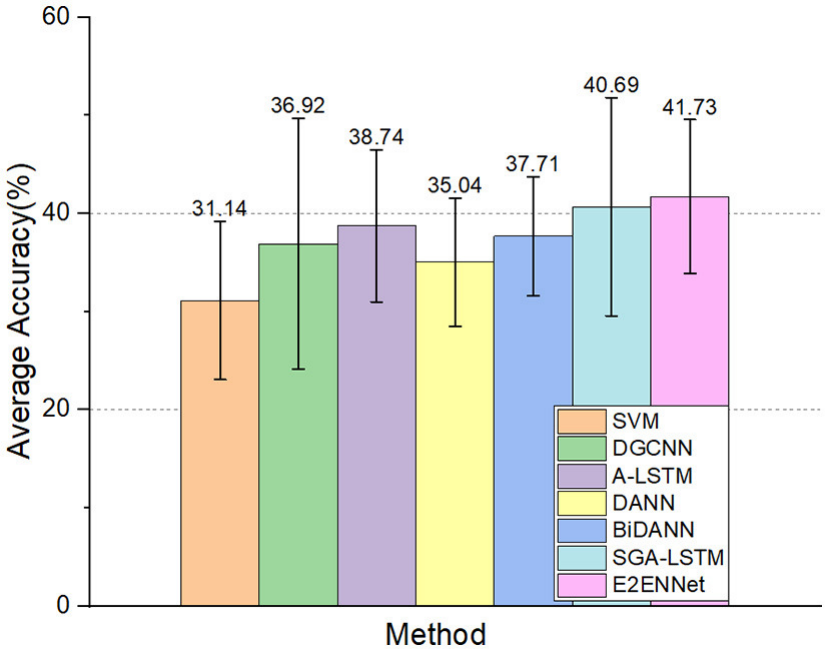
Seven-category classification experiment on MPED dataset.

The experimental results can be seen in Figure 5. The accuracy of the E2ENNet model is still improved compared with other methods. It shows that the E2ENNet model can still maintain a considerable classification effect for more detailed emotion classify circumstances, which fully verifies the good robustness of the E2ENNet model.

### 3.4. Model validation experiments

#### 3.4.1. Influence of different input features

Here, we discuss the influence of different input features for the E2ENNet model. We extract DE and PSD features that refers to Jia et al. (2020b)'s method. The two manually extracted features, the original EEG features (Raw data), and the combination of the three are used as the input data of E2ENNet. We conducted experiments on all three datasets.

The experimental results are shown in Table 3, where ACC represents the recognition accuracy of the model and STD represents the standard deviation. When inputting the manually extracted DE and PSD features, the recognition accuracy of the E2ENNet model is 18.61, 15.95, 9.3, 1.33, 3.89, and 1.23% lower than that of inputting original EEG signals on DEAP, DREAMER and MPED dataset, respectively. The standard deviation is also higher by 1.87, 1.68, 7.75, 0.75, 0.06, and 0.04%, respectively. It shows that some valuable information for emotion classification in original EEG signals may be lost when manually extracting features from EEG signals. At the same time, it also reduces some trainable samples, which also explains the importance of end-to-end emotion recognition. When putting PSD, DE and raw data together. The experimental results show that the results of the three combinations are not as good as the results of using the original signal. The possible reasons is that our network model is to first perform a convolution operation on the original signal, which is equivalent to filtering, and the PSD and DE features are features that have been filtered and then transformed. Therefore, the combined features of the three are not suitable for our network and may lead to redundancy of features, which reduces the effect.

**Table 3:** Experiments on DEAP, DREAMER and MPED datasets of using different input data of E2ENNet.

**Feature**	**DEAP(ACC ± STD)**	**DREAMER(ACC ± STD)**	**MPED(ACC ± STD)**
PSD	77.60 ± 6.05%	88.84 ± 9.18%	37.84 ± 7.90%
DE	80.26 ± 5.86%	96.81 ± 2.18%	40.50 ± 7.88%
Raw data+PSD+DE	77.54 ± 8.80%	89.50 ± 9.04%	38.54 ± 8.68%
**Raw data**	**96.21 ± 4.18%**	**98.14 ± 1.43%**	**41.73 ± 7.84%**

Bold values represent the highest accuracy.

#### 3.4.2. Ablation study

To further verify the necessity of each model module, ablation experiments on E2ENNet were carried out on DEAP, DREAMER, and MPED datasets. It mainly includes the following experiments: (1) Removing EEGNet module in E2ENNet, retain LSTM module only, experiment on original EEG signals; (2) Removing LSTM module in E2ENNet, only retain EEGNet module, experiment on original EEG signals; (3) Experiment on the final E2ENNet model.

**Figure 6:**
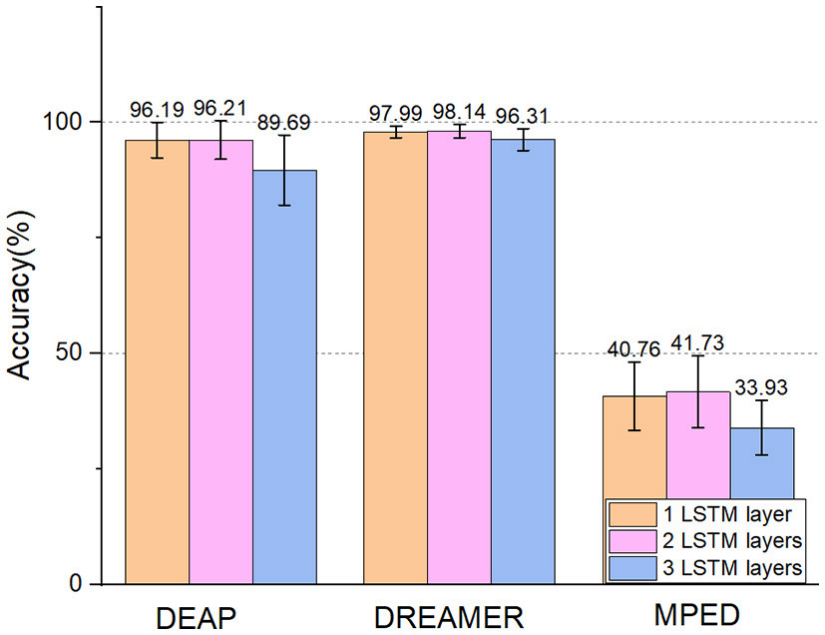
Experiments on DEAP, DREAMER, and MPED dataset based on different LSTM layers in E2ENNet model.

The results can be seen in Table 4, where ACC represents the recognition accuracy of the model and STD represents the standard deviation. And the number of layers of LSTM may influence the classification accuracy, too. We conduct experiments based on one LSTM layer to three LSTM layers. To further verify the effect of different LSTM layers. The results can be seen in Figure 6. We can see that:

- Compared with the E2ENNet model, the recognition accuracy of the LSTM model on DEAP, DREAMER, and MPED dataset is relatively low. This shows that the convolution network EEGNet in E2ENNet, especially the depthwise separable convolution, can extract useful features in original EEG signals and play an important role in emotion recognition.
- After adding the LSTM module to the EEGNet module, the classification accuracy of the E2ENNet model is improved by 1.32, 0.76, and 1.70%, respectively, indicating that LSTM is very sensitive to time-series and can explore helpful pieces of information between features to improve the classification performance of a convolution network.
- Different LSTM layers can influence the effect of E2ENNet. After adding one layer of LSTM to the EEGNet, the effect is improved. After adding two layers, the effect is more obvious. After adding three layers, the effect gradually decreases. Therefore, adding two layers of LSTM makes the emotion recognition model optimal. The reason for the gradual decline after the three-layer LSTM network may be that the EEG emotion data set is a small data set, and too many network layers lead to over fitting.

From the above points, it can be concluded that each part of E2ENNet is effective and has significant contributions to the emotion classification task and the structure of E2ENNet is reasonable.

**Table 4:** Ablation experiments of E2ENNet on DEAP, DREAMER and MPED datasets.

**Model**	**DEAP(ACC ± STD)**	**DREAMER(ACC ± STD)**	**MPED(ACC ± STD)**
E2ENNet(no conv)^a^	63.46 ± 8.05%	82.78 ± 7.34%	32.18 ± 9.29%
E2ENNet(no LSTM)^b^	94.89 ± 6.68%	97.38% ± 1.86%	40.03 ± 7.34%
**E2ENNet** ^c^	**96.21 ± 4.18%**	**98.14 ± 1.43%**	**41.73 ± 7.84%**

E2ENNet(no conv)^a^ denotes the E2ENNet without all convolution blocks, only retain LSTM block; E2ENNet(no LSTM)^b^ denotes the E2ENNet without LSTM block, only retain convolution block. E2ENNet^c^ demotes the complete E2ENNet model, containing all convolution and LSTM blocks. Bold values represent the highest accuracy.

#### 3.4.3. Comparison of time cost

Except for accuracy, computational efficiency (time cost) is also a vital criterion for evaluating algorithms. Due to the effectiveness of depthwise separable convolution we used in E2ENNet, the amount of calculation can be reduced significantly. We run all the codes under the same experimental environment. As shown in Table 5, we compared the training time, testing time, and accuracy of SVM, DBN, CapsNet (Chen et al., 2021) and three ablation models mentioned in Section 3.4.2 on the DEAP dataset. We can see that SVM is the least time-consuming method but the accuracy is not very good, the testing time of DBN is too long. The CapsNet model has relatively high accuracy, but the time cost is very high, too. E2ENNet (no conv) has no advantage in both time cost and accuracy. For E2ENNet (no LSTM) and E2ENNet, the price of higher accuracy is a heavier computation burden during training. However, once the models are trained, we do not need to consider the training time anymore. Our E2ENNet model has achieved relatively the lowest testing cost and the highest accuracy, and run end-to-end, which is very suitable for instant EEG emotion recognition systems.

**Table 5:** The time cost of different models on DEAP dataset.

**Model**	**Training time**	**Testing time**	**ACC**
SVM	**<1s**	**<1s**	75.14%
DBN	/	35s	85.42%
CapsNet	181s	16s	95.33%
E2ENNet(no conv)	252s	8s	63.46%
E2ENNet(no LSTM)	51s	**<1s**	94.89%
**E2ENNet**	72s	**<1s**	**96.21%**

Bold values represent the lowest time cost and the highest accuracy.

## 4. Conclusion

In this paper, we proposed an end-to-end emotion recognition model, E2ENNet, which can extract more discriminative features conductive to emotion recognition from the original EEG signals. Through extensive validation, E2ENNet has achieved state-of-the-art accuracy on three public datasets, i.e., DEAP, DREAMER, and MPED. It's an idea plug-and-pay model for instant emotional brain-computer interface system. At the same time, we noticed that some deeper networks lead to overfitting due to the small EEG samples. In the future, we will use the Generative Adversarial Network to generate EEG data and apply a deeper model to classify emotions.

## Data availability statement

Publicly available datasets were analyzed in this study. This data can be found here: DEAP: http://www.eecs.qmul.ac.uk/mmv/datasets/deap/; MPED: https://github.com/Tengfei000/MPED/.

## Ethics statement

Written informed consent was obtained from the individual(s) for the publication of any potentially identifiable images or data included in this article.

## Author contributions

ZH and HC: conceptualization. HC: methodology. XZ: formal analysis, investigation, and funding acquisition. ZH: resources and data curation and writing—original draft preparation. HC, XZ, JW, LW, and YS: writing—review and editing. All authors have read and agreed to the published version of the manuscript.

## Funding

This work was supported by National Natural Science Foundation of China under Grant 62076064.

## Conflict of interest

The authors declare that the research was conducted in the absence of any commercial or financial relationships that could be construed as a potential conflict of interest.

## Publisher's note

All claims expressed in this article are solely those of the authors and do not necessarily represent those of their affiliated organizations, or those of the publisher, the editors and the reviewers. Any product that may be evaluated in this article, or claim that may be made by its manufacturer, is not guaranteed or endorsed by the publisher.
